# Body Posture, Postural Stability, and Metabolic Age in Patients with Parkinson's Disease

**DOI:** 10.1155/2017/3975417

**Published:** 2017-06-27

**Authors:** Jacek Wilczyński, Agnieszka Pedrycz, Dariusz Mucha, Tadeusz Ambroży, Dawid Mucha

**Affiliations:** ^1^Institute of Physiotherapy, Department of Medicine and Health Sciences, Jan Kochanowski University of Kielce, Kielce, Poland; ^2^Department of Histology and Embryology with Unit of Experimental Cytology, Medical University of Lublin, Lublin, Poland; ^3^Department of Physical Education and Sport, Academy of Physical Education in Cracow, Cracow, Poland

## Abstract

**Background:**

The study aims to analyze the relationship between body posture and composition, as well as postural stability in Parkinson's disease patients.

**Material and Methods:**

32 people were evaluated. The study was conducted in the Laboratory of Posturology at Jan Kochanowski University in Kielce (Poland). Body posture was examined using the optoelectronic body posture Formetric Diers Method III 4D. Postural stability was evaluated using the Biodex Balance System platform. Body composition was assessed with the method of bioelectrical impedance analysis using the Tanita MC 780 MA analyzer.

**Results:**

11 patients (34.37%) had hyperkyphosis, 10 (31.25%) hyperlordosis, and 3 (9.37%) hyperkyphosis-hyperlordosis posture. Scoliosis (>10°) was observed in 28 (87.5%) subjects, whereas 4 (12.5%) presented scoliotic body posture (1–9°). In the examined population, all parameters of postural stability were within normal limits.

**Conclusions:**

A significant positive correlation was observed between surface rotation (°), General Stability Index (*r* = 0.4075, *p* = 0.0206), and Anteroposterior Stability Index (*r* = 0.3819, *p* = 0.0310). There was also a significant positive correlation between surface rotation (+max) (°), General Stability Index (*r* = 0.3526, *p* = 0.0206), and Anteroposterior Stability Index (*r* = 0.3873, *p* = 0.0285). Metabolic age also presented a significant positive correlation between metabolic age and General Stability Index (*r* = 0.4057, *p* = 0.0212), as well as Anteroposterior Stability Index (*r* = 0.3507, *p* = 0.0490).

## 1. Introduction

Parkinson's disease (PD) is one of the most common conditions of the central nervous system [1]. This disease is progressive and results from a loss of cells in the substantia nigra within the mesencephalon [1]. Usually, it begins between the ages of 58 and 62 years; however, cases have also been reported in younger patients [2]. Initially, it was believed that prevalence of PD was identical in both men and women. However, the latest epidemiological studies report that PD is more prevalent in men [3]. Every year in Poland approximately 80.000 people suffer from PD, and the annual morbidity approximates to 8.000 [4]. The neurodegenerative process begins several years prior to the onset of motor symptoms [5]. The disease may develop for many years without any significant clinical symptoms. The search for preclinical markers of PD may enable early treatment and, therefore, delay and alleviate symptoms [6]. The estimated duration of the preclinical phase ranges from 5 to 20 years [7]. During the course of Parkinson's disease, dopaminergic neurons located in the substantia nigra and other dopaminergic regions of mesencephalon die, resulting in a decreased dopamine concentration in the striatum and its progressive degeneration. At an early stage, the putamen and part of the striatum, which mostly receives impulses from motor cortex, are damaged. In the next stages, damage also occurs in the caudate nucleus, which is engaged in cognition, memory, and attention [8]. Clinical diagnosis of Parkinson's disease requires the presence of two out of three main axial symptoms: resting tremor, bradykinesia, and rigidity [9]. Bradykinesia must be present to diagnose PD [10]. The motor symptoms of PD are present in everyday life, such as difficulties in unassisted walking, performing precise activities, beginning the motion, and balance disorders and falls [11]. As PD progresses, the patient develops an abnormal body posture and gait pattern due to rigidity and bradykinesia [12]. The patient flexes the head and cervical spine forward, the thoracic kyphosis increases, lumbar lordosis decreases, both shoulder joints are extended, abducted, and internally rotated, the elbow joints are flexed with pronation, metacarpophalangeal joints are extended, the thumb is abducted, hip joints are slightly adducted, flexed, and internally rotated, and knee joints are slightly flexed [13]. An abnormal posture and scoliosis result from structural damage in the brain regions controlling motor activity, such as basal ganglia as well as nuclei of the thalamus and cerebellum [14].

Postural instability and balance disorders also result from PD [11, 15]. Maintenance of the vertical body posture and its control are complex and consist of mobility and correction processes [16, 17]. Maintaining postural stability is a dynamic process, which is based on contracting various disturbances [18, 19]. These disturbances may be influenced by the external environment as well as internal environment of the human body [20, 21]. Postural stability is affected by progressive and chronic degenerative changes within the structures of CNS that control motor function, the visual system, hearing, proprioception, and balance. Patients become prone to falls, especially during rapid head movements [15, 22]. Disorders in body composition and body weight are also observed as PD progresses [23]. Patients often lose weight, even when their calorific intake is higher than that of healthy individuals at respective ages [24]. Weight changes are also related to gastrointestinal disorders, most likely due to dysfunction of the autonomic nervous system [25, 26]. This study aims to analyze the relationship between body posture, postural stability, and body constitution in patients with Parkinson's disease.

## 2. Materials and Methods

A group of 32 patients was examined, who were members of the Parkinson Disease Association in Kielce (Poland). The majority was females, 26 (81.25%), and there were 6 males (18.75%). The study was conducted in November 2013 in the Laboratory of Posturology at the Institute of Physiotherapy of the Faculty of Medicine and Health Sciences, Jan Kochanowski University in Kielce. All patients were treated with levodopa (L-DOPA). This is an endogenous amino acid, catecholamine, produced by tyrosine hydroxylation and catalysed by tyrosine hydroxylase. Levodopa is the precursor of dopamine, which causes an increased concentration of this neurotransmitter in the brain. The treatment with L-DOPA was designed as one of the criteria for inclusion into the study. The patients participated in structured physiotherapy course for a period of 12 months. All procedures performed in studies involving human participants were in accordance with the ethical standards of the institutional and/or national research committee and with the 1964 Helsinki declaration and its later amendments or comparable ethical standards. The patients were informed of the objective of the study and expressed written consent to participate in this study. The study was noninvasive and free of charge. The patients willingly participated in the study and perceived it as concerns about their state of health. Body posture was examined by the optoelectronic test of body posture, the Formetric Diers Method III 4D using raster stereography. Three-dimensional analysis of the spine was achieved by combination of modern optical techniques and digital data processing. This is a quick and touchless photometric 4D measurement and analysis of the patient's back surface and the spine. The following parameters describing body posture were analyzed:

1. Kyphotic angle ICT-ITL (max°)* (kyphotic angle inflexion point cervical thoracic- inflexion point thoracic lumbar max*°): this is the maximum kyphotic angle measured between the tangents to the surface of the upper cervical thoracic inflexion point (ICT), in the vicinity of the vertebra prominens (VP), and thoracic lumbar inflexion point (ITL).
2. Lordotic angle ITL-ILS (max°)* (lordotic angle inflexion point thoracic lumbar- inflexion point lumbar-sacral max*°): this is the maximum lordotic angle measured between the tangents to the surface of the thoracic lumbar inflexion point ITL and the lower lumbar-sacral inflexion point ILS.
3. Scoliosis angle °: measurement with Diers formetric III 4D concerns exclusively the spine and shows the curvature angle from 1°.
4. Pelvic tilt in degrees (°): pelvic tilt refers to the difference in the height of sacral dimples DL-DR* (dimple left-dimple right)*, in relation to the transverse surface (cross-section). A positive value means that the right dimple is higher than the left dimple, whereas a negative value occurs when the right dimple is located below the left dimple.
5. Pelvic tilt (mm): pelvic tilt refers to the difference in heights of the sacral dimples DL-DR* (dimple left-dimple right)*, in relation to the transverse surface (cross-section). A positive value means that the right dimple is higher than the left dimple, whereas a negative value occurs when the right dimple is located below the left dimple.
6. Surface rotation max°: this parameter defines the maximum rotation of the surface of the vertebrae on a symmetry line. Positive values mean the maximum surface rotation is to the right, while negative values mean maximum surface rotation is to the left.
7. Surface rotation (+max.)°: this parameter defines the maximum right-side rotation of the surface of the vertebrae on the symmetry line to the right.

Postural stability was evaluated using the Biodex Balance System platform. Postural Stability Test was performed with both feet positioned on a stable background, with open eyes. The platform was blocked, which means that it was rigid and fully stable. Application of the patients' personal data and body height allowed their position to be determined. This allowed the centre line of the foot and platform axes to be used as the points of reference. The position was determined by entering on the screen of the device the angles of the position of the feet, using the centre line separately for the right and left foot. The Postural Stability Test consisted of three 20-second trials, each separated by 10-second breaks. During the test, the patient's vision was focused on a characteristic dot (COP, centre of pressure) on the monitor screen, which was a symbolic presentation of the centre of feet pressure. The patient's task was to balance the body in such a way that the dot (COP) was in the centre of a circle displayed on the monitor at the point of intersection of the coordinate axes. During the examination, verbal correction of the patient was permitted. All the parameters registered by the posturological platform were collected completely noninvasively, and the device was deemed safe for the patients recruited to the study. For assessment of postural stability, the following measurements were used:

- Overall Stability Index (°) reflects variability of the positioning of the platform with respect to the horizontal plane, expressed in degrees during all movements performed in the test. Its high value evidences a large amount of movements performed during the test.
- Anterior-Posterior Stability Index (°) reflects variability of the platform displacement in the sagittal plane, expressed in degrees.
- Medial-Lateral Stability Index (°) reflects variability of the platform displacement in the frontal plane, expressed in degrees.

The patient's scoring in the Postural Stability Test depended on the number of sways from the centre. This meant that the lower the result, the better the postural stability. The percentage of time in zone (%) index is the time spent by a patient in any given zone. Target zones A, B, C, and D are equal with respect to the degree of platform tilt. They are determined by concentric circles with the middle in the centre of the platform: Zone A, from zero to five degrees of deviation with respect to the horizontal plane; Zone B, from six to ten degrees of deviation with respect to the horizontal plane; Zone C, from eleven to fifteen degrees of deviation with respect to the horizontal plane; Zone D, from sixteen to twenty degrees of deviation with respect to the horizontal plane; and Time in Quadrant (%). This index is the time which the patient spent in any given quadrant. Quadrants represent four quadrants of the test graph between* y*- and* x*-axis: Quadrant 1, right anterior; Quadrant 2, left anterior; Quadrant 3, left posterior; and Quadrant 4, right posterior.

Body composition was assessed using the method of bioelectrical impedance analysis (BIA), which consists of evaluating the resistance of flow of an electric current. For the BIA analysis, knowledge is used concerning the prevalence of electrolytes and better electrical conductivity of muscle tissue, which contains a considerable amount of water; in turn, adipose tissue is less conductive. The BIA is a reliable, noninvasive, and easily available means for the estimation of body composition parameters. For this research, a body composition analyzer was used, Tanita MC 780 MA. The results of the measurements allowed the following variables to be obtained: body mass (kg), body mass index, metabolic age, fat mass (%), fat mass (kg), fat-free mass (kg), muscle mass (kg), visceral fat, total body water (kg), and total body water (%). The variables were compared in terms of sex using the Mann–Whitney test. The relationship between body posture, postural stability, and body composition was analyzed using Spearman's rank correlation coefficient. The probability *p* < 0.05 was considered significant.

## 3. Results

The analysis of anthropometric variables revealed significant differences between females and males in terms of height (*Z* = 3.7541, *p* = 0.0002) and body mass (*Z* = 3.2592, *p* = 0.0011), whereas there were no differences with regard to BMI (*Z* = 1.4735, *p* = 0.1406) and metabolic age (*Z* = 0,2175, *p* = 0,8278). Significant differences in body composition between females and males referred to fat-free mass (kg) (*Z* = 3.1627, *p* = 0.0016), muscle mass (kg) (*Z* = 3.7421, *p* = 0.0002), visceral fat (*Z* = 1.9892, *p* = 0.0467), total body water (kg) (*Z* = 3.7428, *p* = 0,0002), and total body water (%) (*Z* = 2.8257, *p* = 0.0047). Only the fat mass (kg) was similar (*Z* = 0.5795, *p* = 0.5622) (Table 1). Three-dimensional evaluation of the spine revealed hyperkyphosis (łac.* dorsum rotundum*) in 11 patients (34.37%), hyperlordosis (łac.* dorsum concavum*) in 10 (31.25%), and hyperkyphosis-hyperlordosis posture (łac.* dorsum rotundo-concavum*) in 3 (9.37%). Scoliosis (łac.* scoliosis*) (>10°) was detected in 28 (87.5%) individuals, whereas 4 patients (12.5%) had scoliotic posture (1–9°). Left-lateral pelvic tilt was detected in 11 (34.37%) subjects and right-lateral in 12 (37.5%). Left surface rotation was detected in 19 (59.37%) patients and right in 13 (4.62%). Right surface rotation (+max) was found in 27 (84%) patients and 5 (16%) did not present with any rotation. Significant differences in body posture between females and males included scoliotic angle (°) (*Z* = 2.1271, *p* = 0.0334), chest length (VP-DM) (mm) (*Z* = 2.9939, *p* = 0.0027), and chest length (VP-SP) (mm) (*Z* = 2,8249, *p* = 0,0047). There were no differences in terms of body posture within the following variables: thoracic kyphosis angle (°) (*Z* = 1.2572, *p* = 0.2087), scoliosis angle (°) (*Z* = 1.3066, *p* = 0.1914), pelvic tilt (°) (*Z* = 1.3754, *p* = 0.1690), pelvic tilt (mm) (*Z* = 1.3789, *p* = 0.1679), vertebral surface rotation (max°) (*Z* = 1.1377, *p* = 0.2552), and surface rotation (+max) (°) (*Z* = 1.1181, *p* = 0.2635) (Table 2). The Postural Stability Test revealed significant differences between females and males in Overall Stability Index (°) (*Z* = 2,0545, *p* = 0,0399). Higher Overall Stability Index in females indicated slightly lower postural stability compared to males. The variables of postural stability which did not significantly differ include Anterior-Posterior Index (°) (*Z* = 1.3825, *p* = 0.1668), Medial-Lateral Index (°) (*Z* = 1.7927, *p* = 0.0730), % Time in A Zone (*Z* = 0,6330, *p* = 0,5267), % Time in B Zone (none of the participants of the study was in C and D Zones), % Time in Quadrant I (0.0242, *p* = 0.9807), % Time in Quadrant II (*Z* = 0.4875, *p* = 0.6259), % Time in Quadrant III (*Z* = 0.6541, *p* = 0.5131), % and Time in Quadrant IV (%) (*Z* = 0.0000, *p* = 1.0000). All parameters of postural stability were within normal limits in the study group. More significant postural inclinations were observed in the sagittal plane than in frontal plane. During testing, most of the patients were slightly bended backwards and were in quadrant IV (Table 3).

## 4. Discussion

Postural and body composition disorders are important symptoms of PD as a chronic neurological disability. In our study, 11 (34.37%) of patients with PD had hyperkinetic hyperosmolarity (hyperkyphosis), 10 (31.25%) had enlarged lumbar ligaments (hyperlordosis), and 3 persons (9.37%) had simultaneous deep thoracic kyphosis and lumbar lordosis (hyperkyphosis-hyperlordosis). Scoliosis (>10°) was observed in 28 (87.5%) patients, and in 4 (12.5%), scoliosis posture (1–9°) occurred. In their studies, other authors also observed changes in body posture in PD patients with excessive neck flexion (NF), excessive bending of the knee joints (KB), lateral bending (LB), and forward bending (FB). The angle of neck flexion was greater in men than women; the remaining changes in posture showed a significant difference between the sexes. The forward tilting of the body and the knee flexion increased with age. Excessive flexion of the neck and forward tilting of the body were associated with the duration of the disease. All changes in posture significantly increased with the development of the disease. The forward inclination angle of the body was significantly related to the levodopa dosage, but dopamine agonist doses did not show a significant correlation with the change in posture [27]. In other studies conducted among Parkinson's patients, scoliosis has been reported, but more commonly in women. There was also a positive relationship between the severity of Parkinson's disease symptoms and the extent of scoliosis [28, 29]. In our study, all parameters of postural stability in patients with PD were normal. Greater postural sways occurred in the sagittal plane compared to the frontal plane. At the time of the test, most of the subjects were slightly inclined backwards and remained within square IV. In other studies, however, changes in postural control in the form of muscle stiffness as well as motor slowdown in corrective and anticipatory reactions have been observed. Sensory integration disorders, slowing of gait, freezing, and decreasing automation of gait and balance were also noted. Furthermore, it has been shown that levodopa does not have a positive effect on postural disorders, and, therefore, patients require rehabilitation [30]. In the research by other authors, it has been shown that patients with PD have significant postural stability limitations, lower scores in the LOS (limits of stability), lower values of functional balance reserves, and greater postural sways compared to healthy individuals. The deterioration of posture control was strongly associated with a high risk of falls [31]. In another study, it was analyzed whether postural stabilization responses can be improved and preserved among PD patients in which the slowdown leads to freezing in a manner similar to that used in people without freezing. Both individuals with and without freezing symptoms did not improve their results nor were there permanent changes to the postural response system with respect to the main variable, centre of mass (COM). However, other gait-protection related results, including stability margin, length, and pace, improved at similar levels in all groups. Significant improvements were maintained in both groups. In conclusion, individuals with PD who have a tendency to halt movement have decreased ability to improve postural protective responses to some but not all variables [32]. In our study, the vast majority of PD patients' body composition parameters were normal, and metabolic age was significantly lower than calendar age. The results confirm the proper planning and course of treatment as well as the proper nutrition of the subjects. In other similar studies among PD patients, rarely were the subjects underweight, and the risk of malnutrition was common but stable. However, the value for the arm skin fold increased. There was a decrease in muscle circumference in the upper half of the arm. The percentage of individuals with poor handshake pressure increased. Correlations between dietary and motor variables and nonmotor PD characteristics were from scarce to moderate. In addition, increased anxiety was correlated with weight loss, BMI, and arm skin folds value. As the disease progressed, redistribution in the body structure from muscle to fat occurred [18]. In a different study, the body composition of PD patients was analyzed three years after diagnosis. Based on the method of electrical impedance, a slight increase in body mass was noted. It was strongly correlated with increased body fat, waist circumference, body height to waist circumference ratio, and total skin fold. In this work, we also demonstrate the relationship between body mass changes and the level of physical activity and the relationship between body mass changes and mental health (Mini Mental State Examination). At the onset of the disease, weight gain was accompanied by an increase in waist circumference and body mass. The opposite correlation was observed for body mass index and physical activity level [33]. Another objective of the study was the prospective assessment of body fat and its distribution in patients with PD. The distribution of adipose tissue in PD patients was compared with healthy subjects of similar age using magnetic resonance imaging. MRIs were conducted for 12 months. Total body fat volume as well as visceral fat did not show any significant differences between PD patients and healthy subjects, regardless of the moment of measurement. However, in PD patients, a decrease in the volume of subcutaneous adipose tissue was observed, as well as a higher ratio of visceral fat to subcutaneous fat compared to the control group. It has been reported that 16 patients did not lose weight, while 9 patients lost from 0.5 to 10 kg. Fat distribution was altered and the ratio of visceral to subcutaneous fat increased [34]. In our study, there were also significant positive correlations between metabolic age and the Overall Stability Index (*r* = 0.4057, *p* = 0.0212) and Front-Back Stability Index (*r* = 0.3507, *p* = 0.0490) (Table 4). The higher metabolic age was associated with the higher Overall Stability Index and higher Front-Back Stability Index. There were also significant positive correlations between surface rotation (°) and the Overall Stability Index (*r* = 0.4075, *p* = 0.0206) and the Front-Back Stability Index (*r* = 0.3819, *p* = 0.0310) (Table 4). The higher surface rotation (°) results were associated with the higher scores of the Overall Stability Index and Front-Back Stability Index. There were also significant positive correlations between the surface rotation (+max) (°) and the Overall Stability Index (*r* = 0.3526, *p* = 0.0206) and the Front-Back Stability Index (*r* = 0.3873, *p* = 0.0285) (Table 4). Higher surface rotation (+max) (°) results were associated with the higher results of the Overall Stability Index and Front-Back Stability Index. The changes in body posture, postural stability, and body composition are detrimental from the perspective of maintaining the health of a Parkinson's disease patient, and therefore, systematic research and prevention are of importance.

## 5. Conclusions

1. Most of the studied patients had scoliosis. Scoliotic postures, pelvic tilling, vertebral surface rotation, hyperkyphosis, hyperlordosis, hyperkyphosis-hyperlordosis, and flat-back postures were also observed.
2. All parameters of postural stability in the study group were within the normal limits. More significant postural inclinations were observed in the sagittal plane compared to the frontal plane. During testing, most patients tended to bend backwards towards quadrant IV. Monitoring of postural stability in patients with PD is important due to the risk of falls in this group. The results of postural stability testing suggest that most patients received appropriate treatment and physical therapy.
3. Most parameters of body composition were within the normal range in the study group, and metabolic age was significantly lower compared to calendar age. These results indicate that the patients' treatment was planned well, as they received appropriate treatment and adequate nutrition.
4. There were significant positive correlations between surface rotation (°), Overall Stability Index, and Anterior-posterior Stability Index. There were also significant correlations between surface rotation (+max) (°), Overall Stability Index, and Anterior-posterior Stability Index. The metabolic age significantly correlated positively with the Overall Stability Index and Anterior-posterior Stability Index.

## Figures and Tables

**Table 1:** Anthropometric variables and body composition.

Anthropometric variables	Gender	Arithmetic mean	Standard deviation	Minimum	Lower quartile	Median	Upper quartile	Maximum	Mann–Whitney *U* test
Body height (cm)	Total	165.5	8.10	150.0	159.5	164.5	170.2	184.0	*Z* = 3.7541 *p* = 0.0002
	Females	162.6	5.78	150.0	158.0	164.0	166.7	172.0	
	Males	177.8	3.71	174.0	175.2	177.0	179.5	184.0	

Body weight (kg)	Total	66.14	11.05	48.30	58.10	65.60	74.05	89.00	*Z* = 3.2592 *p* = 0.0011
	Females	62.74	8.79	48.30	57.35	59.35	71.50	78.20	
	Males	80.85	7.15	71.50	75.55	81.15	86.75	89.00	

BMI	Total	24.12	3.49	17.50	21.65	23.10	26.00	32.30	*Z* = 1.4735 *p* = 0.1406
	Females	23.78	3.66	17.50	21.35	22.35	25.78	32.30	
	Males	25.57	2.41	22.90	23.58	25.35	27.43	28.70	

Metabolic age (MA)	Total	42.34	11.74	20.00	34.00	43.00	49.25	70.00	*Z* = 0.2175 *p* = 0.8278
	Females	42.35	12.03	20.00	34.00	43.00	49.00	70.00	
	Males	42.33	11.45	27.00	35.00	42.50	51.50	55.00	

Fat mass (%)	Total	27.14	6.35	16.80	22.23	26.45	32.28	38.10	*Z* = 3.1627 *p* = 0.0016
	Females	28.80	5.80	17.70	24.25	28.30	33.70	38.10	
	Males	19.95	2.39	16.80	18.53	20.25	20.63	23.70	

Fat mass (kg)	Total	18.04	5.55	9.50	14.10	15.90	21.78	29.80	*Z* = 0.5795 *p* = 0.5622
	Females	18.46	5.95	9.50	14.10	15.90	23.20	29.80	
	Males	16.23	3.03	12.50	14.53	15.70	17.63	21.10	

FFM (kg)	Total	48.10	9.01	36.70	42.65	45.30	49.08	70.10	*Z* = 3.7417 *p* = 0.0002
	Females	44.28	3.92	36.70	42.43	44.65	46.75	52.70	
	Males	64.62	4.86	57.10	62.05	65.45	67.88	70.10	

Muscle mass (kg)	Total	45.65	8.58	34.80	40.45	43.00	46.55	66.60	*Z* = 3.7421 *p* = 0.0002
	Females	42.02	3.73	34.80	40.23	42.35	44.35	50.00	
	Males	61.38	4.63	54.20	58.95	62.20	64.48	66.60	

Visceral fat	Total	6.69	3.28	2.00	4.00	6.00	8.25	14.00	*Z* = 1.9892 *p* = 0.0467
	Females	6.00	2.71	2.00	4.00	6.00	7.75	12.00	
	Males	9.67	4.08	5.00	6.25	10.00	13.00	14.00	

TBW (kg)	Total	34.00	6.19	26.30	30.20	32.15	34.80	49.20	*Z* = 3.7428 *p* = 0.0002
	Females	31.40	2.79	26.30	29.98	31.60	32.95	37.50	
	Males	45.25	3.41	40.50	43.08	45.45	47.83	49.20	

TBW (%)	Total	51.53	4.48	43.90	47.88	52.30	54.95	58.50	*Z* = 2.8257 *p* = 0.0047
	Females	50.50	4.28	43.90	47.13	50.75	53.85	58.50	
	Males	56.02	1.87	53.10	55.05	56.20	57.50	58.00	

**Table 2:** Variable postures of the body.

Variables of posture	Gender	Arithmetic mean	Standard deviation	Minimum	Lower quartile	Median	Upper quartile	Maximum	Mann–Whitney *U* test
Kyphotic angle (°)	Total	52.47	9.45	30.00	45.25	54.00	59.25	69.00	*Z* = 1.2572 *p* = 0.2087
	Females	51.42	9.45	30.00	43.75	52.00	57.75	69.00	
	Males	57.00	8.72	43.00	52.75	58.50	63.50	66.00	

Lordotic angle (°)	Total	42.75	10.44	17.00	36.75	41.50	49.25	63.00	*Z* = 2.1271 *p* = 0.0334
	Females	44.27	10.78	17.00	40.00	44.50	50.75	63.00	
	Males	36.17	5.56	26.00	34.75	38.00	39.75	41.00	

Scoliotic angle (°)	Total	18.16	9.76	3.00	12.00	16.00	20.25	48.00	*Z* = 1.3066 *p* = 0.1914
	Females	19.35	10.19	5.00	12.25	16.50	22.50	48.00	
	Males	13.00	5.73	3.00	11.75	14.00	15.50	20.00	

Trunk length (VP-DM) (mm)	Total	445.0	40.72	327.0	424.7	445.5	468.0	542.0	*Z* = 2.9939 *p* = 0.0027
	Females	435.5	37.11	327.0	423.2	435.0	460.5	491.0	
	Males	486.2	29.86	464.0	468.7	472.5	492.0	542.0	

Trunk length (mm)	Total	500.9	40.51	377.0	478.2	501.5	521.0	592.0	*Z* = 2.8249 *p* = 0.0047
	Females	492.1	37.92	377.0	474.5	489.0	514.2	584.0	
	Males	538.8	29.25	514.0	520.2	528.0	547.0	592.0	

Pelvic tilt (°)	Total	0.31	4.17	−9.00	−2.00	0.00	2.00	11.00	*Z* = 1.3754 *p* = 0.1690
	Females	−0.19	4.08	−9.00	−2.00	0.00	2.00	11.00	
	Males	2.50	4.18	−2.00	0.00	1.00	5.00	9.00	

Pelvic tilt (mm)	Total	0.38	5.89	−12.0	−3.00	0.00	3.00	15.00	*Z* = 1.3789 *p* = 0.1679
	Females	−0.35	5.65	−12.0	−3.00	0.00	3.00	15.00	
	Males	3.50	6.41	−3.00	0.00	1.50	5.25	15.00	

Surface rotation (°)	Total	−0.34	9.72	−13.0	−8.00	−4.00	9.00	16.00	*Z* = 1.1377 *p* = 0.2552
	Females	0.73	10.15	−13.0	−8.00	−4.00	9.75	16.00	
	Males	−5.00	6.23	−11.0	−7.75	−6.50	−5.25	7.00	

Surface rotation (+max.) (°)	Total	5.59	5.20	0.00	1.00	4.00	9.25	16.00	*Z* = 1.1181 *p* = 0.2635
	Females	6.27	5.46	0.00	1.00	6.00	11.50	16.00	
	Males	2.67	2.42	1.00	1.00	1.50	3.50	7.00	

**Table 3:** Variables of postural stability.

Postural stability variables	Gender	Arithmetic mean	Standard deviation	Minimum	Lower quartile	Median	Upper quartile	Maximum	Mann–Whitney *U* test
Overall Stability Index (°)	Total	0.50	0.35	0.20	0.30	0.40	0.60	1.90	*Z* = 2.0545 *p* = 0.0399
	Females	0.55	0.37	0.20	0.30	0.50	0.60	1.90	
	Males	0.30	0.13	0.20	0.20	0.25	0.38	0.50	

Anterior-Posterior Stability Index (°)	Total	0.35	0.24	0.10	0.20	0.30	0.40	1.30	*Z* = 1.382 *p* = 0.1668
	Females	0.37	0.26	0.10	0.20	0.30	0.40	1.30	
	Males	0.23	0.10	0.10	0.20	0.20	0.28	0.40	

Medial-Lateral Stability Index (°)	Total	0.27	0.25	0.10	0.10	0.20	0.30	1.10	*Z* = 1.7927 *p* = 0.0730
	Females	0.30	0.26	0.10	0.10	0.20	0.30	1.10	
	Males	0.13	0.05	0.10	0.10	0.10	0.18	0.20	

Zone A (%)	Total	99.94	0.25	99.00	100.0	100.0	100.0	100.0	*Z* = 0.6330 *p* = 0.5267
	Females	99.92	0.27	99.00	100.0	100.0	100.0	100.0	
	Males	100.0	0.00	100.0	100.0	100.0	100.0	100.0	

Zone B (%)	Total	0.09	0.30	0.00	0.00	0.00	0.00	1.00	*Z* = 0.8125 *p* = 0.4165
	Females	0.12	0.33	0.00	0.00	0.00	0.00	1.00	
	Males	0.00	0.00	0.00	0.00	0.00	0.00	0.00	

Zone C (%)	Total	0.00	0.00	0.00	0.00	0.00	0.00	0.00	*Z* = 0 *p* = 0
	Females	0.00	0.00	0.00	0.00	0.00	0.00	0.00	
	Males	0.00	0.00	0.00	0.00	0.00	0.00	0.00	

Zone D (%)	Total	0.00	0.00	0.00	0.00	0.00	0.00	0.00	*Z* = 0 *p* = 0
	Females	0.00	0.00	0.00	0.00	0.00	0.00	0.00	
	Males	0.00	0.00	0.00	0.00	0.00	0.00	0.00	

Quadrant 1 (%)	Total	19.72	16.05	0.00	5.75	15.00	33.00	64.00	*Z* = −0.0242 *p* = 0.9807
	Females	19.50	16.10	0.00	6.25	14.50	33.00	64.00	
	Males	20.67	17.33	2.00	6.50	20.00	29.75	47.00	

Quadrant 2 (%)	Total	5.81	9.29	0.00	1.00	3.00	5.25	46.00	*Z* = 0.4875 *p* = 0.6259
	Females	6.31	10.24	0.00	1.00	2.00	6.00	46.00	
	Males	3.67	1.97	0.00	3.25	4.50	5.00	5.00	

Quadrant 3 (%)	Total	12.94	11.83	0.00	3.00	10.00	17.50	41.00	*Z* = 0.6541 *p* = 0.5131
	Females	12.65	12.28	0.00	3.00	9.00	16.75	41.00	
	Males	14.17	10.59	3.00	5.50	14.50	18.25	31.00	

Quadrant 4 (%)	Total	61.53	22.25	11.00	44.75	62.50	76.75	96.00	*Z* = 0.0000 *p* = 1.0000
	Females	61.54	23.64	11.00	45.50	66.00	78.00	96.00	
	Males	61.50	16.56	42.00	48.75	61.00	72.50	84.00	

**Table 4:** Correlation between metabolic age, surface rotation of the vertebrae, and postural stability variables.

Statistical variables	Overall Stability Index (°)	Anterior-Posterior Stability Index (°)	Medial-Lateral Stability Index (°)
Metabolic age			
Spearman correlation coefficients (*r*)	0.4057	0.3507	0.1529
Standard error of the (*r*)	0.1669	0.1710	0.1804
95% CI^1^ in (*r*)			
0.0556	−0.0084	−0.2171	−0.3776
0.6669	0.6298	0.4845	0.3384
*t*-test for significance of the (*r*)	2.4312	2.0515	0.8477
Degree of freedom (df)	30	30	30
*p* value	**0.0212** ^2^	0.0490^2^	0.4033

Surface rotation (°)			
Spearman correlation coefficients (*r*)	0,4075	0.3819	0.2443
Standard error of the (*r*)	0.1667	0.1687	0.1770
95% CI in (*r*)			
0.0578	0.0275	−0.1247	−0.2171
0.6681	0.6510	0.5540	0.4845
*t*-test for significance of the (*r*)	2.4443	2.2632	1.3801
Degree of freedom (df)	30	30	30
*p* value	0.0206^2^	0.0310^2^	0.1777

Surface rotation (+max) (°)			
Spearman correlation coefficients (*r*)	0.3526	0.3873	0.1433
Standard error of the (*r*)	0.1708	0.1683	0.1807
95% CI in (*r*)			
−0.0063	0.0338	−0.2264	−0.2171
0.6311	0.6546	0.4770	0.4845
*t*-test for significance of the (*r*)	2.0640	2.3009	0.7932
Degree of freedom (df)	30	30	30
*p* value	0.0478^2^	0.0285^2^	0.4339

^1^Confidence interval (CI).

^2^Statistically significant at *p* < 0.05.
